# A Case Report of Vulvar Leiomyoma: A Rare Pathological Entity

**DOI:** 10.7759/cureus.42878

**Published:** 2023-08-02

**Authors:** Minal Kalambe, Preeti Gattani, Nandkishor J Bankar

**Affiliations:** 1 Obstetrics and Gynaecology, Datta Meghe Medical College, Datta Meghe Institute of Higher Education and Research, Wardha, IND; 2 Microbiology, Jawarhal Nehru Medical College, Datta Meghe Institute of Higher Education and Research, Wardha, IND

**Keywords:** labia minora, bartholin cysts, vulvar mass, vulvar lesions, labial leiomyoma

## Abstract

Smooth muscle tumors, known as leiomyomas, are benign and can grow anywhere in the body, but the uterine myometrium is the most common site. Vulvar leiomyomas are uncommon and rare. We report a case of a 25-year-old female P (para) 1 L (live) 1 with a previous normal vaginal delivery, who presented two painless swelling of in vulva for one year. The urethral meatus deviated to the left side. After surgical excision, a histopathological examination was performed that confirmed leiomyoma. The patient was discharged after an uneventful postoperative period. A vulvar leiomyoma tumor is often mistaken for a Bartholin cyst, and it is challenging to distinguish between benign and malignant types of vulvar leiomyomas, making diagnosis extremely challenging.

## Introduction

Leiomyomas of the uterus are the most common gynecologic neoplasms frequently encountered in females of reproductive age. Up to 30% of females may develop uterine leiomyoma, benign tumors arising from smooth muscle cells. They can occur in any organ system that contains smooth muscle, including the gastrointestinal tract, the genitourinary tract, and the respiratory system [1]. Although leiomyomas are the most frequently benign compact neoplasm of the vulva, they are still relatively uncommon. Vulvar leiomyomas represent only 0.03% of all gynecological neoplasms and 0.07% of all vulvar tumors [2].

Smooth muscle tumors are unusual and sometimes mistaken clinically as Bartholin cysts. The labia majora, blood vessel walls, round ligament, the dartos muscle, and erector pili muscle are thought to be the origin of these tumors. Although extremely rare, valvular leiomyomas have been found in the literature, misdiagnosed as Bartholin cysts [3]. We present a case of vulvar leiomyoma in a young female.

## Case presentation

**Figure 1:**
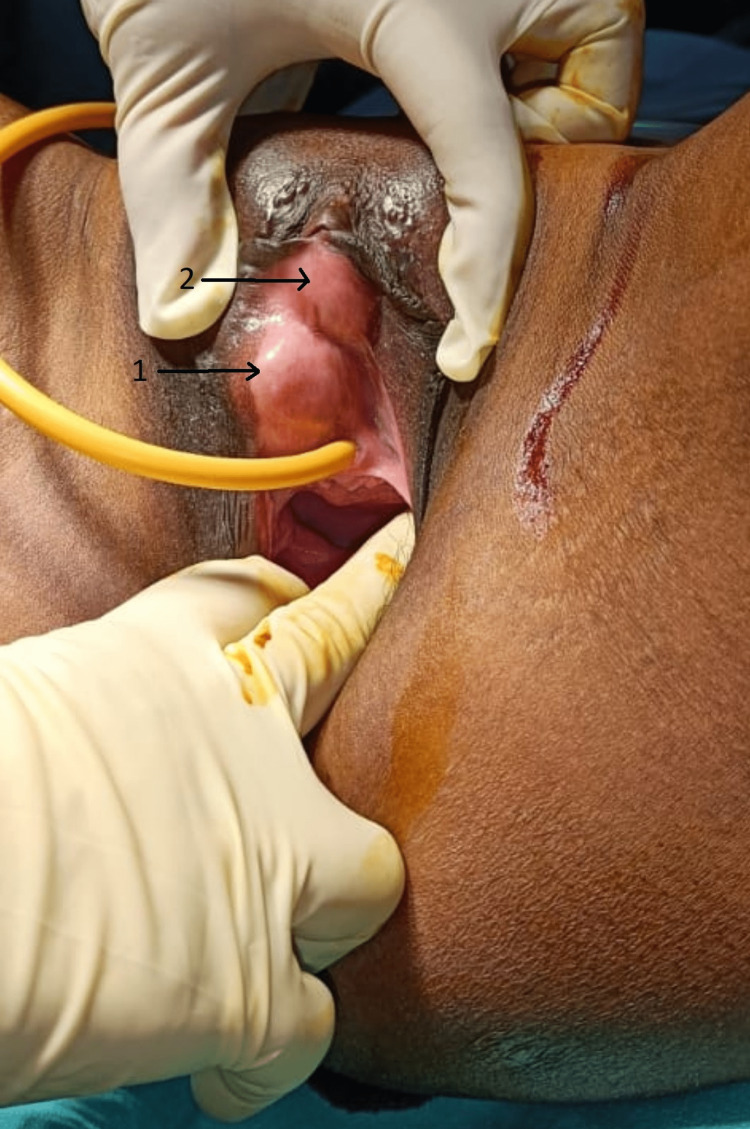
Vulvar Leiomyoma (Before Surgery) Arrow indicates the two leiomyomas.

A 25-year-old, P1 (para one) L1 (live one) female, came to our outpatient clinic complaining of a painless swelling on her left vulva, which was growing in size. Her menstrual cycles were regular, painless, and not associated with clots. She had no significant medical history or surgical history. There was no history of vaginal discharge, difficulty in micturition, or fever, and her bowel habits were regular. She denied any history of sexual trauma, dyspareunia, or other vulvar pathology and had no significant medical history. On examination, a firm, well-defined, non-tender mass was noted in the left valvar region. She appeared to be in fair physical condition, and her physiological parameters were constant. She was afebrile, and there was no inguinal lymphadenopathy. On local examination, the clitoris appeared normal; the urethral meatus deviated to the left side. The patient was catheterized easily, and the labia majora and labia minora were easily separated. On the left side of the labia minora, the first mass/swelling was observed, 1 cm x 3 cm x 2 cm, and the second mass/swelling observed just above the first was 1 cm x 2 cm x 1 cm, which has keratinization on the anterior surface and was stable, not reducible, and painless (Figure 1). On per speculum examination, the cervix and vagina were healthy, and on per vaginal examination, the uterus was anteverted, average-sized, and bilaterally fornix-free and non-tender.

**Figure 2:**
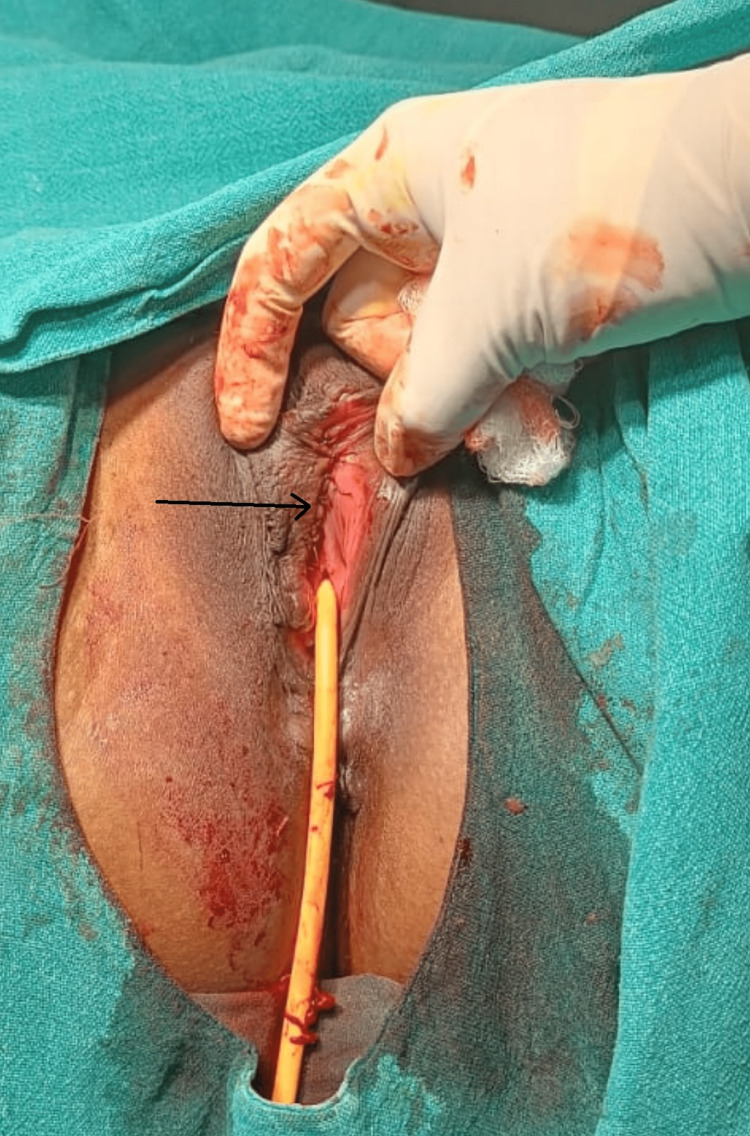
Vulva After Surgery Arrow indicates suturing of the surgical site.

The overlying skin was normal in appearance. Ultrasound examination of the pelvis was normal. The size was average and anteverted, with a normal endometrial lining, and both ovaries were normal. Magnetic resonance imaging (MRI) of the pelvis was normal. Two palpable masses in the left labium minora, measuring 2 cm x 3 cm x 2 cm and 1 cm x 2 cm x 1 cm, were observed. These masses were well-circumscribed and hypoechoic and did not show vascularity on Doppler imaging. A provisional diagnosis of a vulvar leiomyoma was made, and the surgical excision of the mass was planned under spinal anesthesia. The incision was made on the anterior surface of the tumor, and then enucleation was done. The mass had a whorl-like appearance on the cut section, and the surgical area was sutured (Figure 2).

**Figure 3:**
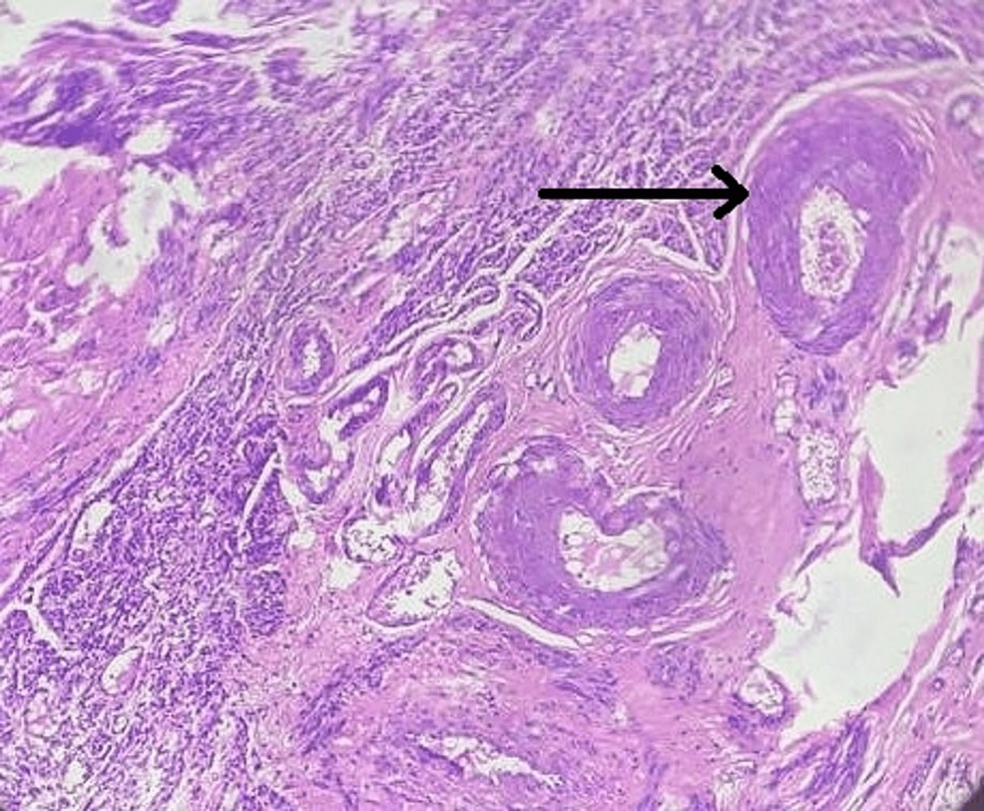
Histopathology of the Leiomyoma Arrow shows the spindle cells of the leiomyoma.

The postoperative recovery of the patient was uneventful. Histopathological examination of the excised mass confirmed the diagnosis of a leiomyoma. The tumor was composed of interlacing bundles of smooth muscle cells (Figure 3).

 

## Discussion

A limited number of vulvar leiomyomas have been reported in the literature, making them sporadic tumors [3]. They usually arise from smooth muscle cells of the vulvar or the dartos muscle and are solitary, well-circumscribed, and slow-growing lesions. The exact etiology of vulvar leiomyomas is unknown, but they are believed to arise from pluripotent mesenchyme cells that differentiate into smooth muscle cells. The rare tumor, known as vulvar leiomyomas, is frequently detected in females of reproductive age. Due to their rarity, these tumors morphological and epidemiological characteristics of these tumors cannot be fully understood [3,4]. The deep connective tissue of the introitus, labia majora, perineal body, round ligament, and stem cells in the Bartholin gland may give rise to the tumor.

The ovaries, bladder, urethra, sinonasal cavities, orbits, and kidneys are unusual locations for leiomyomas [4]. Initially, leiomyomas are asymptomatic. They start to show symptoms when they grow and develop complications, such as superficial ulceration. Leiomyomas are asymptomatic but can expand to enormous sizes. They may have an unusual growth pattern or manifest in an unusual area, making identification difficult [5]. The differential diagnosis includes soft tissue sarcoma, Bartholin cyst, fibroma, lymphangioma, and neurogenic tumor. Ultrasound is the most effective and popular diagnostic method for uterine and extrauterine conditions. Most tumors are solitary, well-defined masses. Symptoms include sitting discomfort, difficulty urinating, pain, and difficulty walking [6,7].

The patient may have anxiety and cosmetic problems due to the size and appearance of the tumor. When cancer is symptomatic, surgical excision of the tumor is a possible treatment [8,9]. The leiomyomas and capsule must be removed entirely to reduce the likelihood of recurrence. Abdomino-peritoneal approaches are preferred when treating giant tumors. A subsequent microscopic examination can typically reveal its structure, validating the diagnosis [10,11]. Surgical excision is the treatment of choice for vulvar leiomyoma, and the prognosis is excellent, with no reported cases of malignant transformation or recurrence after complete excision [12,13].

## Conclusions

We present a case of vulvar leiomyoma in a young female. A vulvar leiomyoma tumor is mistaken mainly for Bartholin's cyst, and it is challenging to distinguish between benign and malignant types of vulvar leiomyomas, making the diagnosis extremely difficult. The procedure currently used, excisional biopsy, is the most effective. After therapy, a follow-up is necessary if there is any difficulty in that region.
